# A Craniopharyngioma Associated With Elevated Cerebrospinal Fluid HCG Concentrations Misdiagnosed as a Germinoma

**DOI:** 10.3389/fneur.2018.00449

**Published:** 2018-06-14

**Authors:** Weijun Gu, Weizhong Gu, Yulin Gu, Jie Li, Guoqing Yang, Qinghua Guo, Li Zang, Jin Du, Yu Pei, Jianming Ba, Zhaohui Lv, Jingtao Dou, Yiming Mu

**Affiliations:** ^1^Department of Endocrinology, Chinese People's Liberation Army General Hospital, Beijing, China; ^2^Department of Neurology, The Second People's Hospital of Datong, Shanxi, China; ^3^Department of Pathology, Chinese People's Liberation Army General Hospital, Beijing, China

**Keywords:** craniopharyngioma, germinoma, human chorionic gonadotrophin, pituitary neoplasms, cranial tumors

## Abstract

Craniopharyngiomas and germinomas are both rare cranial tumors that most commonly present during childhood or adolescence. Although these tumors have different origins, their clinical and radiological features may be similar. In this article, we report the case of a 35-year female patient with clinical and radiological findings and increased human chorionic gonadotrophin (HCG) levels in the cerebrospinal fluid (CSF) that were consistent with a germinoma. However, pathological analysis revealed a craniopharyngioma. This case report indicates that HCG, which is regarded as a specific tumor marker for germinomas in the differential diagnosis of intracranial lesions, is also detectable in other kinds of suprasellar tumors, such as craniopharyngiomas.

## Introduction

Craniopharyngiomas are rare benign tumors derived from the cellular remnants of Rathke's pouch, along a line from the nasopharynx to the diencephalon. Epidemiological studies have demonstrated that craniopharyngiomas account for ~3% of all intracranial tumors. Craniopharyngiomas are the most common form of non-neuroepithelial neoplasm in the pediatric population (1). Although these tumors are histologically benign, craniopharyngiomas are often large and tend to behave aggressively, infiltrating the surrounding brain structures. Treatment mainly involves surgical resection. The early establishment of a diagnosis is important for optimum treatment and a successful outcome. However, despite the characteristic findings associated with these tumors, discrimination between craniopharyngiomas and other intracranial tumors is clinically and radiologically difficult (2). In this report, we present the case of a female patient with a suprasellar craniopharyngioma associated with a thickened pituitary stalk without calcification and increased human chorionic gonadotrophin (HCG) concentrations in the cerebrospinal fluid (CSF). The relevant literature is reviewed. Informed consent was obtained from the patient.

## Case report

**Figure 1:**
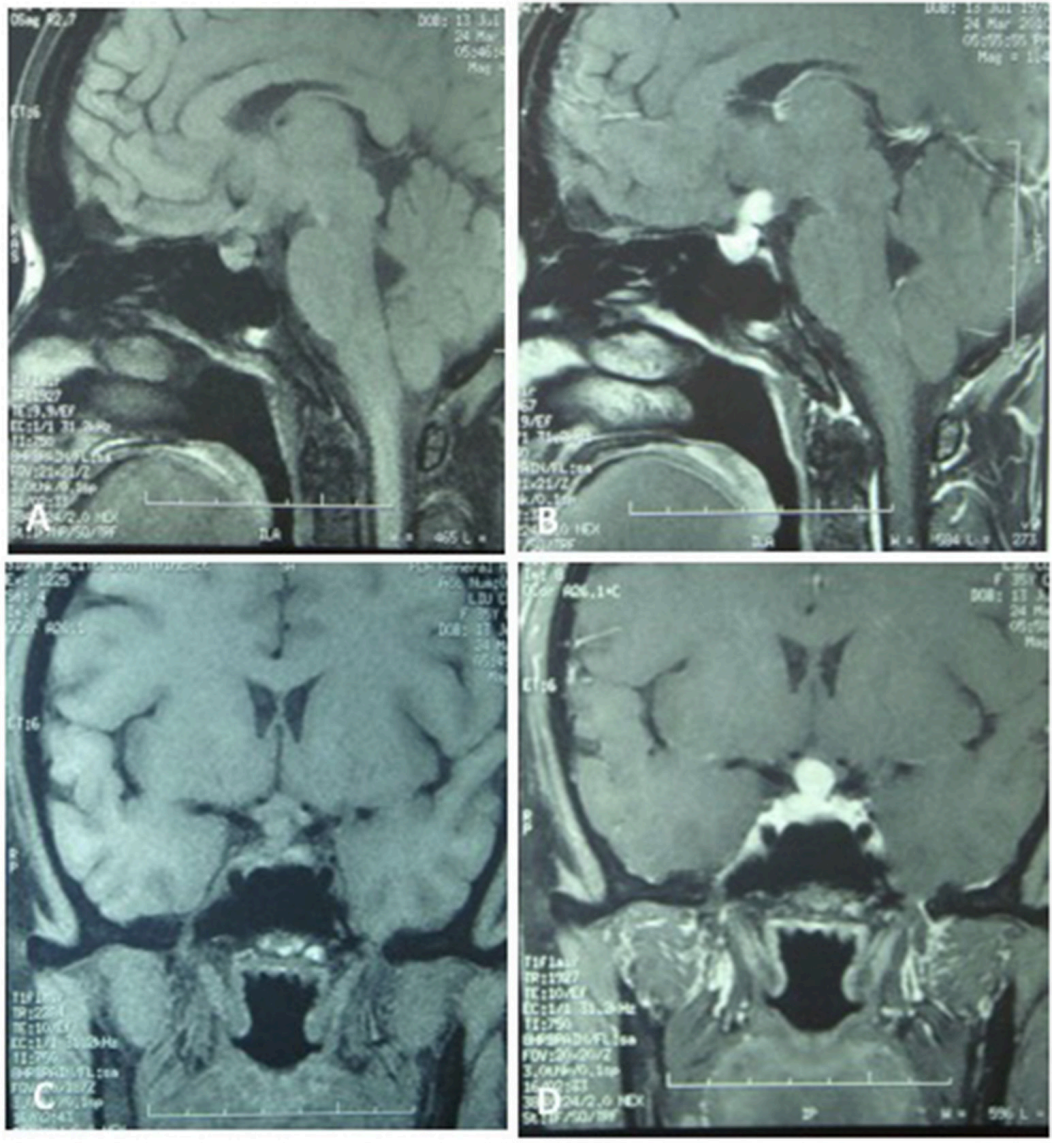
The MRI of the brain and the pituitary. **(A,C)** No significant expansion of the sella, irregular cystic-solid mixed mass in the hypothalamus region, solid part presented iso-signal, the boundaries between the lesion and pituitary stalk, pituitary are not clear, the optic chiasm was compressed. **(B,D)** The solid part of the lesion was significantly enhanced, no enhancement in cystic part, no abnormal signal in the brain parenchyma and paranasal sinus.

**Figure 2:**
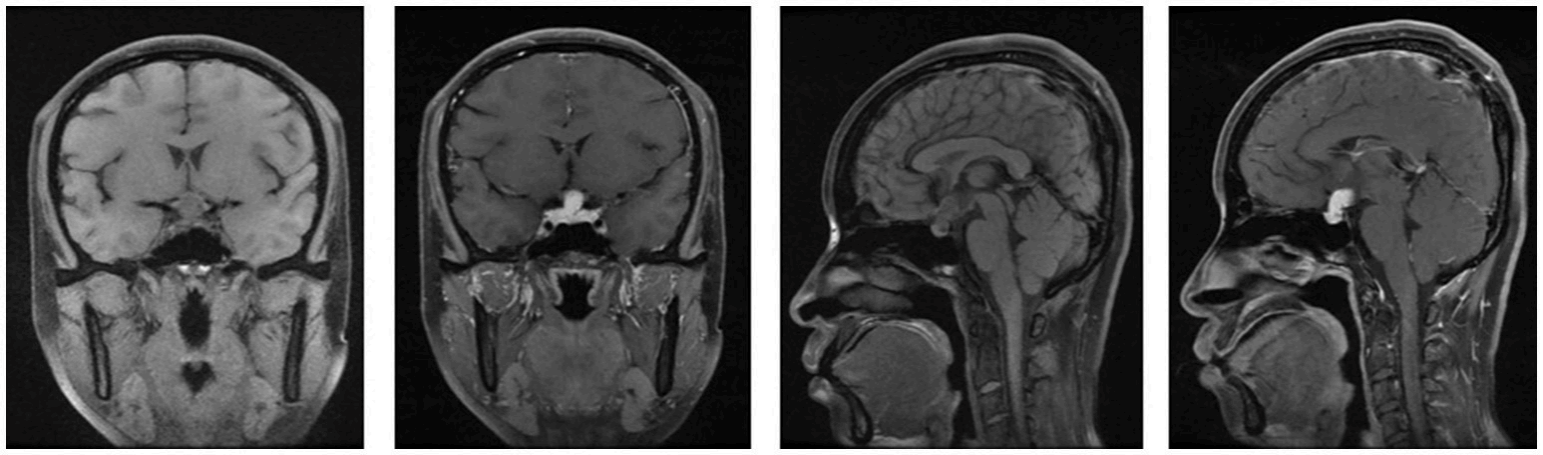
The MRI of brain and pituitary (second hospitalization). No signs of shrinkage for the mass.

**Figure 3:**
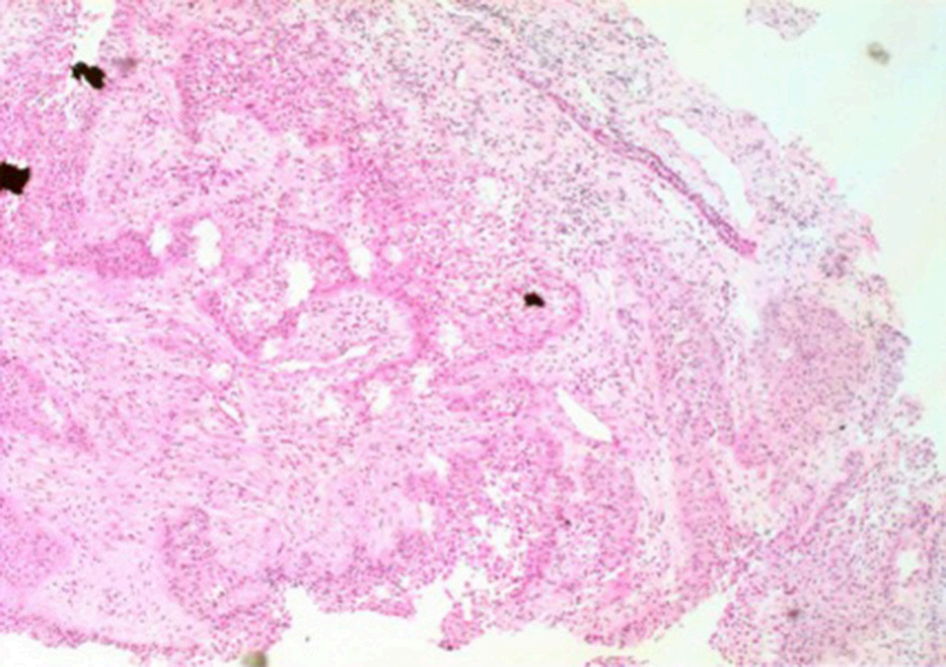
The image of pathology. Palisaded columnar epithelium cells, stellate reticular cells, and keratinized tissues are dominant, and glial hyperplasia zone is adjacent to the nerve tissue, compatible with adamantinomatous craniopharyngioma (X 200).

A 35-year-old female was admitted to our hospital complaining of an intermittent headache for 5 years, menopause for more than 2 months, and blurred vision and lactation for more than 1 month. Five years prior, the patient began suffering from intermittent and gradually deteriorating occipital and frontal headaches. One month prior to admission, she had impaired vision and an absent right temporal view and simultaneous bilateral galactorrhea, without purulent or bloody secretions. Since the onset of symptoms, the patient experienced no nausea or vomiting, sleepiness, chills, hair loss, or fatigue. Computed tomography (CT) showed an irregular cystic solid mass in the suprasellar area. The size of the mass was 1.4^*^1 cm and could be markedly enhanced. The CT value of the solid part of the lesion on plain CT was 40 Hounsfield units (HU); in the enhancement, the value was 74 HU, and the cystic part showed no enhancement. The boundary of the lesion was less clear, the density was not uniform, and no obvious calcification was observed. The cavernous sinus was involved. There were no abnormalities in the ventricular system, sulcus, brain split and pool. Moreover, there was no shift in the midline structure, and no abnormalities were found in the skull structure. The sinuses exhibited no obvious abnormalities. Magnetic resonance imaging (MRI) of the brain and pituitary showed an irregular cystic solid mass in the suprasellar area, involving in the pituitary stalk, and the boundary between the lesion and pituitary was unclear. In addition, the mass pressed on the optic chiasm, and the solid part of the lesion was significantly enhanced, while the cystic part showed no enhancement. There was no abnormal signal in the brain or paranasal sinuses nor signs of calcification (Figure 1). Auxiliary examinations revealed that the patient's urine volume and specific gravity were within the normal range (urine volume: 1,800 mL/day; urine specific gravity: 1.015). A pituitary function evaluation showed hyperprolactinemia (45.47 μg/L, normal range for female: 2.8–29.2 U/L) and intact growth hormone (GH) and gonadotropin function. The insulin-like growth factor 1 (IGF-1) level was 120 ng/ml (109–284 ng/ml). The pituitary-thyroid and pituitary-adrenal axis were intact (Tables 1–4). The blood sedimentation and CRP level were normal, and indicators related to tuberculosis and other infections were negative. Because of the thickening of the pituitary stalk, CSF analysis was performed to identify the nature of the mass lesion before any invasive procedures were performed. The results of CSF analysis were normal except for a significantly increased human HCG (18.54 U/L, normal range: 0–5 U/L) level. However, the serum HCG and α-fetoprotein (AFP) levels were normal. A biopsy was recommended to clarify the diagnosis, but the patient declined. Considering the possibility of a germinoma, radiotherapy was recommended. No regression of the intracranial mass and no resolution of the headache were observed after 10 radiotherapy treatments (total 10 Gy). Therefore, the patient was admitted a second time. Apart from a deficiency in GH and hypogonadotropic hormones, the other axes were intact (Tables 5–7). The IGF-1 level was 89 ng/ml (109–284 ng/ml). A repeated CSF analysis indicated there was no obvious decrease in the HCG level in the CSF (17.41 U/L, normal range: 0–5 U/L). An MRI showed no signs of shrinkage of the mass (Figure 2). Due to the enlarged intracranial mass, the patient was transferred to the neurosurgical department for total resection of the mass. The pathological analysis was indicative of a craniopharyngioma (Figure 3).

**Table 1:** Circadian rhythm of ACTH, cortisone (first hospitalization).

	**0 a.m**.	**8 a.m**.	**4 p.m**.
ACTH (pmol/L)	5.7	6.4	<2.2
F (nmol/L)	363	709	203
UFC (nmol/24 h)	463.4		

*F, cortisol*.

*Reference range: ACTH: 8 a.m.: ACTH < 10.12 pmol/l*.

*F: 8 a.m.: 198.7–797.5 nmol/l, 4 p.m.: 85.3–459.6 nmol/l, 0 a.m.: 0–165.7 nmol/l*.

*UFC: 98.0–500.1 nmol/24 h*.

**Table 2:** Thyroid Function: Results show the intact of thyroid function.

**Time**	**TSH (mU/L)**	**T4 (nmol/L)**	**T3 (nmol/L)**	**FT4 (pmol/L)**	**FT3 (pmol/L)**	**TgAb (IU/mL)**	**TPOAb (IU/mL)**
BF	3.238	90.2	1.72	15.27	4.35	348.6	27.5
AF	4.229	87.1	1.59	14.66	4.06	52.3	45.4

*F, cortisol; BF, before the surgery; AF, after the surgery*.

*Reference range: TSH: 0.35–5.5 mU/L; T4: 55.34–160.88 nmol/L; T3: 1.01–2.95 nmol/L; FT4: 10.42–24.32 pmol/L; FT3: 2.76–6.3 pmol/L; TgAb: <60 IU/ml; TPOAb: <60 IU/ml*.

**Table 3:** Insulin hypoglycemia stimulation test (first hospitalization): Results show the intact of growth hormone.

**Time(min)**	**Glucose (mmol/L)**	**ACTH (pmol/L)**	**F (nmol/L)**	**GH (ug/L)**
0	5.09	3.3	384.2	0.76
30	2.04	20.5	625.8	2.11
60	12.59	15.2	762.2	15.7
90	7.1	6.3	642.9	9.32

*F, cortisol*.

**Table 4:** LH and FSH value (basal and response to intravenous injection of 100 ug gonadotropin-releasing hormone) (first hospitalization): Results show the intact of gonadotropin function.

	**−15 min**	**0 min**	**30 min**	**60 min**	**120 min**
LH (IU/L)	2.42	1.85	19.76	17.02	11.28
FSH (IU/L)	7.69	7.00	22.17	23.04	22.06

**Table 5:** Circadian rhythm of ACTH, cortisone (second hospitalization): Results show that the pituitary-adrenal axes was intact.

	**0 a.m**.	**8 a.m**.	**4 p.m**.
ACTH (pmol/L)	2.2	6.7	4.0
F (nmol/L)	44.99	476.19	235.5
UFC (nmol/24 h)	395.6		

*F, cortisol*.

*Reference range: ACTH: 8 a.m.: ACTH < 10.12 pmol/l*.

*F: 8 a.m.: 198.7–797.5 nmol/l, 4 p.m.: 85.3–459.6 nmol/l, 0 a.m.: 0–165.7 nmol/l*.

*UFC: 98.0–500.1 nmol/24 h*.

**Table 6:** Insulin hypoglycemia stimulation test (second hospitalization): Results show the deficiency of growth hormone.

**Time(min)**	**Glucose (mmol/L)**	**ACTH (pmol/L)**	**F (nmol/L)**	**GH (ug/L)**
0	4.43	3.55	206.17	0.48
30	1.28	19.9	409.92	1.09
60	14.25	26.6	571.28	5.19
90	3.40	9.28	555.84	2.71

*F, cortisol*.

**Table 7:** LH and FSH value (basal and response to intravenous injection of 100 ug gonadotropin-releasing hormone) (second hospitalization): Results show the deficiency of gonadotropic hormones.

	**−15 min**	**0 min**	**30 min**	**60 min**	**120 min**
LH (IU/L)	0.59	0.55	9.03	8.46	5.60
FSH (IU/L)	5.15	5.02	20.67	23.60	20.80

### Surgical approach

Under a microscope, the lateral fissure was separated at the saddle area, and CSF was released. The brain tissue collapsed, and a portion of the temporal lobe was removed. The tumor was located in the suprasellar area. The tumor was removed from the second gap along the longitudinal axis of the pituitary stalk and was frozen for analysis. The tumor and hypothalamus were closely related. Hemostasis was achieved, and the gauze inventory was correct. The surgery was complete following strict closure of the dura mater, bone flap reduction and fixation, suturing of the temporalis muscle and scalp, and placement of an external dura mater drain. The tumor specimens were sent for pathological analysis.

### Follow-up

**Figure 4:**
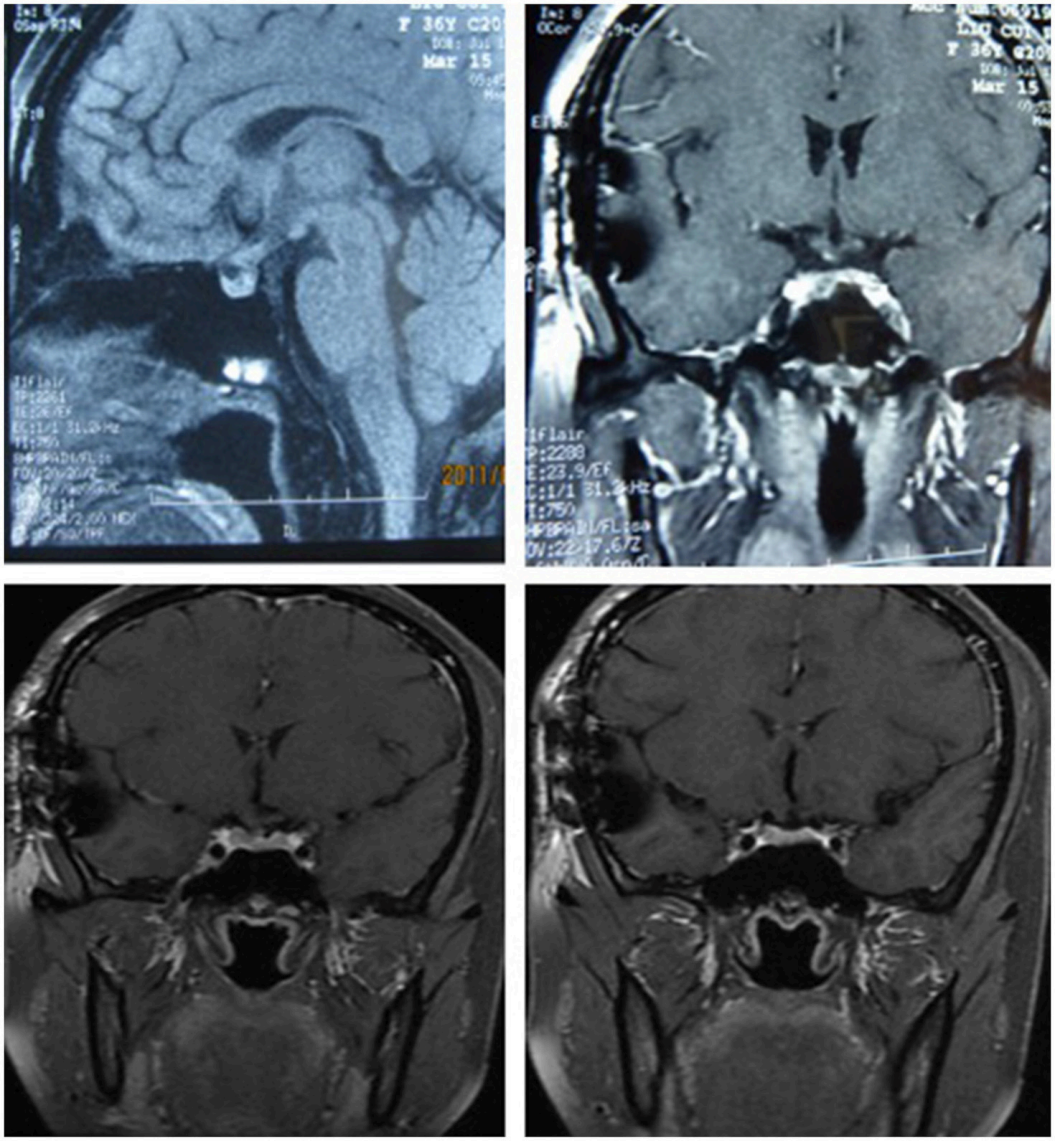
The MRI of cranial and pituitary (1 year after surgery). No expansion of sella region, no signs of local tumor, no abnormal enhancement, clear boundary of optic chiasm, no signs of tumor recurrence.

After surgery, the patient did well and had no complaints of headache, vomiting, polyuria, polydipsia, sleepiness, or anorexia. She did not receive any hormone replacement. One year later, she was admitted to our endocrinology department for re-evaluation. Her urine volume and specific gravity were within the normal range (urine volume: 1,500 mL/day; urine specific gravity: 1.017). The urine osmotic pressure was 752 mOsm/L (0–1,000 mOsm/L), the blood osmolality was 292 mOsm/L, and a pituitary stimulation test revealed normal frontal lobe function except for an impaired GH level (Tables 8, 9). The IGF-1 level was 92 ng/ml. HCG levels in the serum and CSF were within normal limits (serum HCG: 0.1 U/L; CSF HCG: 0.31 U/L). The slightly increased prolactin detected preoperatively had decreased to within the normal range. Cranial and pituitary MRI showed no expansion of the sellar region, no signs of a local tumor, no abnormal enhancement, a clear boundary of the optic chiasm, and no signs of tumor recurrence (Figure 4).

**Table 8:** LH and FSH value (basal and response to intravenous injection of 100 ug gonadotropin-releasing hormone) (1 year after surgery): The stimulating test of the pituitary revealed reserved gonadotropic hormones.

	**−15 min**	**0 min**	**30 min**	**60 min**	**120 min**
LH (IU/L)	1.86	2.51	29.64	25.22	15.37
FSH (IU/L)	5.96	7.18	16.40	17.24	16.29

**Table 9:** Insulin hypoglycemia stimulation test (1 year after surgery): The stimulating test of the pituitary revealed the deficiency of growth hormone.

**Time(min)**	**Glucose (mmol/L)**	**ACTH (pmol/L)**	**F (nmol/L)**	**GH (ug/L)**
0	4.74	4.6	150.9	0.43
30	2.23	11.2	305.4	0.62
60	4.93	18.2	589.4	0.37
90	3.85	7.3	358.9	5.73

*F, cortisol*.

## Discussion

Craniopharyngioma may occur at any age but most commonly presents during childhood or adolescence. Craniopharyngioma has two prevalence peaks, one at 5–14 years of age and another at 50–74 years of age. Compared with the general population, adult-onset craniopharyngioma patients have much higher mortality, specifically, up to 19-fold higher cerebrovascular mortality (3). Most craniopharyngiomas are detected in the sellar/parasellar region, but rare ectopic locations have also been described, including in the pineal gland, cerebellopontine angle, temporal lobe, or completely within the 3rd ventricle. Craniopharyngioma is the most common suprasellar tumor in the pediatric age group. The tumor size varies between small, solid, well-circumscribed masses and large multilobular cysts. The consistency of craniopharyngiomas may be solid, cystic, or a combination of cystic and solid (4). Calcification, a typical characteristic, occurs in ~60% of cases (probably more common in children) (5). The clinical manifestations are variable and depend on the tumor location, size, growth pattern, and relationship to adjacent cerebral structures. In adults, the most frequent presenting clinical symptoms are visual field deficits and signs of hypopituitarism. Compared with childhood-onset craniopharyngioma patients, adult-onset craniopharyngioma patients present much more pronounced hormonal deficits at the time of diagnosis. Disturbances in the hypothalamic–pituitary axis affect GH secretion (75%), gonadotropins (40%), adrenocorticotropic hormone (ACTH) (25%), and thyroid-stimulating hormone (TSH) (25%). At the time of diagnosis, 40–87% of patients present with at least one hormonal deficit (6). A craniopharyngioma is suspected mainly based on clinical and radiological findings. The combination of solid, cystic, and calcified tumor components is an important radiological clue for the diagnosis. The differential diagnosis includes a number of sellar or parasellar lesions, such as Rathke's cleft cyst, dermoid cyst, epidermoid cyst, pituitary adenoma, germinoma, hamartoma, suprasellar aneurysm, arachnoid cyst, suprasellar abscess, glioma, meningioma, sarcoidosis, tuberculosis, and Langerhans cell histiocytosis (7). Clinically, these tumors are not easy to distinguish from other tumors because of their non-specific features, such as headache, hypopituitarism, or visual disturbances. Despite the characteristic MRI findings, it is still difficult to differentiate these tumors from pituitary adenomas and germinomas (8). In a previous report, a young girl with a mixed germ cell tumor in the pituitary-hypothalamic region presented with clinical and radiological features resembling a craniopharyngioma (9).

Intracranial germinomas account for 1–3% of all primary intracranial tumors in Western countries but 4–10% in East Asia (10). Similar to craniopharyngiomas, germinomas can also affect the suprasellar region, resulting in the same clinical manifestations. Intracranial germinomas most frequently occur in children and young adults (10). There are different gender ratios depending on the location of the tumor. Neurohypophyseal germinomas develop more frequently in female patients than male patients (11). Moreover, intracranial germinomas also share the same radiological features as craniopharyngiomas and are characterized by high density on unenhanced CT and non-specific T1 hypointensity and T2 isointensity to hyperintensity with cystic changes and homogeneous or heterogeneous enhancement (12). Compared with other intracranial tumors, intracranial germinomas are more sensitive to radiotherapy and have the potential to be cured. Therefore, it is crucial to differentiate between intracranial germinomas and other intracranial tumors. Due to the diverse, non-specific clinical manifestations and lack of radiographic characteristics of these tumors, it is challenging to make a precise diagnosis during the initial period. Germinomas contain syncytiotrophoblastic giant cells that secrete HCG. For this reason, HCG is regarded as a tumor marker for these types of tumors. The diagnosis of intracranial germinomas mainly depends on the levels of tumor markers, such as HCG and AFP, in the serum and CSF (13, 14). Radiotherapy is recommended based on high AFP or/and HCG levels in the serum or CSF. This view has many supporters who propose that tumor markers measured in the serum or CSF may replace the need for a biopsy and reduce the consequent elevated risk of infection during neurosurgery.

The combination of the clinical condition of this young woman, who complained of impaired vision and a mild anterior pituitary disturbance, with an intracranial tumor found at the midline, atypical radiological findings and an obviously increased CSF tumor marker (HCG) rendered the diagnosis of germinoma quite reasonable. A HCG level in CSF above 50 pg/ml and a CSF-to-serum HCG ratio of 2.0 or greater are strong indicators of germinoma (15). Since our patient matched these criteria, a cranial germinoma was deemed to be a possible diagnosis. Treatment of intracranial germinomas mainly relies on radiotherapy. Considering that the patient declined a biopsy, radiotherapy was recommended. However, the patient had no response to radiotherapy, and the CSF HCG level remained very high, indicating that this diagnosis was improbable. Finally, a craniopharyngioma was confirmed by biopsy. Cases of craniopharyngioma with high CSF tumor markers (HCG) are very rare in the worldwide literature. Several reported cases have shown that craniopharyngiomas may co-exist with other germ cell tumors, and according to the view of dysembryogenesis, craniopharyngiomas may be one more element of this phenomenon and present with high HCG and AFP levels (16–18). In this case, histological findings excluded the co-occurrence of a craniopharyngioma and germinoma. Even though the disease is sensitive to chemo-radiotherapy, it still has a poor prognosis. In this case, radical surgery enabled complete remission of the clinical condition and tumor marker elevations. At present, the patient has not experienced relapse. HCG is physiologically produced by the placental syncytiotrophoblasts of the placenta to maintain pregnancy. HCG is not a specific placental hormone and can also be found in trophoblastic-derived lesions that are often present in germinoma tissue. HCG as well as its subunits can be detected in the sera of healthy individuals (19). In addition to trophoblastic lesions, elevated HCG can be encountered in other pathologies. HCG positivity has been shown in a variety of malignancies (20). The validation of HCG as a tumor marker has been reported in a previous study (21). Among intracranial lesions, because elevated HCG is found in the CSF or serum in germinomas, it has been suggested that HCG is a specific marker for germinomas that may aid in the differential diagnosis of intracranial lesions (22). Histological verification has been considered unnecessary in the presence of HCG positivity (23). However, the specificity of HCG as a marker for intracranial germinomas has been questioned. Harris and colleagues detected elevated HCG levels in cystic fluid and CSF from a patient with a craniopharyngioma (24). Jurgen Honegger measured HCG and subunits of HCG in cystic fluid, CSF, and serum of patients with intracranial cysts. They found that HCG was markedly elevated in the cystic fluid from all 17 craniopharyngiomas (range 367–4,558 IU/I; normal <5 IU/I), moderately elevated in the cystic fluid from three of four pituitary adenomas, and elevated in two metastases from lung cancer and two arachnoid cysts. No HCG was found in cystic gliomas, meningiomas, or hemangioblastomas. An elevated HCG level in the CSF was detected in only two patients with craniopharyngiomas, but no HCG immunoreactivity was detected in any serum samples. Subtle immunostaining of epithelial cell groups was identified in 5 out of 10 craniopharyngiomas. Clear immunostaining for HCG was also found in scattered epithelial cells from one pituitary adenoma (25). A recent electron microscopic study demonstrated secretory granules in the cytoplasm of epithelial cells, suggesting a secretory component (26). This mechanism might explain the accumulation of HCG immunoreactivity in craniopharyngiomas. The results indicate that HCG is detectable not only in choriocarcinomas and germinomas containing syncytiotrophoblast-secreting cells but also in other kinds of suprasellar tumors.

In conclusion, the clinical features and radiological findings of suprasellar tumors may be similar in both craniopharyngiomas and intracranial germinomas. Although tumor marker measurements in the serum or CSF are helpful for the diagnosis of germ cell tumors and have replaced the need for a biopsy in many cases, the absolute differentiation of atypical germinomas from rare craniopharyngiomas with an elevated HCG level in the CSF or serum is often not possible prior to invasive therapeutic or diagnostic procedures. This information is paramount in guiding the clinician to select the best procedure and avoid misdiagnosis.

## Ethics statement

This patient provided written consent agreeing to undergo treatment and allow the publication of the information that was described in this case report.

## Author contributions

WJG treated this patient and collected the data and was a major contributor in writing the manuscript. WJG and WZG have contributed equally to this work. JL performed the histological examination. YG, GY, QG, LZ, JD, YP, JB, and ZL have done some assisted work. JTD and YM were the superior advisors. All authors read and approved the final manuscript.

### Conflict of interest statement

The authors declare that the research was conducted in the absence of any commercial or financial relationships that could be construed as a potential conflict of interest.
